# The ubiquitin thioesterase YOD1 ameliorates mutant Huntingtin induced pathology in *Drosophila*

**DOI:** 10.1038/s41598-023-49241-8

**Published:** 2023-12-11

**Authors:** Anita Farkas, Nóra Zsindely, Gábor Nagy, Levente Kovács, Péter Deák, László Bodai

**Affiliations:** 1 Department of Biochemistry and Molecular Biology, Faculty of Science and Informatics, University of Szeged, Közép Fasor 52, 6726 Szeged, Hungary; 2 Doctoral School in Biology, Faculty of Science and Informatics, University of Szeged, 6726 Szeged, Hungary; 3 Department of Genetics, Faculty of Science and Informatics, University of Szeged, Közép Fasor 52, 6726 Szeged, Hungary; 4 Divison of Biology and Biological Engineering, California Institute of Technology, 1200 East California Boulevard, Pasadena, 91125 USA

**Keywords:** Genetics, Molecular biology, Neuroscience, Neurology

## Abstract

Huntington’s disease (HD) is a neurodegenerative disorder caused by a dominant gain-of-function mutation in the *huntingtin* gene, resulting in an elongated polyglutamine repeat in the mutant Huntingtin (mHtt) that mediates aberrant protein interactions. Previous studies implicated the ubiquitin–proteasome system in HD, suggesting that restoring cellular proteostasis might be a key element in suppressing pathology. We applied genetic interaction tests in a *Drosophila* model to ask whether modulating the levels of deubiquitinase enzymes affect HD pathology. By testing 32 deubiquitinase genes we found that overexpression of *Yod1* ameliorated all analyzed phenotypes, including neurodegeneration, motor activity, viability, and longevity. *Yod1* did not have a similar effect in amyloid beta overexpressing flies, suggesting that the observed effects might be specific to mHtt. *Yod1* overexpression did not alter the number of mHtt aggregates but moderately increased the ratio of larger aggregates. Transcriptome analysis showed that *Yod1* suppressed the transcriptional effects of mHtt and restored the expression of genes involved in neuronal plasticity, vesicular transport, antimicrobial defense, and protein synthesis, modifications, and clearance. Furthermore, *Yod1* overexpression in HD flies leads to the upregulation of genes involved in transcriptional regulation and synaptic transmission, which might be part of a response mechanism to mHtt-induced stress.

## Introduction

Accumulation of misfolded proteins is a hallmark of several neurodegenerative diseases^1^. In Huntington’s disease (HD, OMIM #143100), a late-onset monogenic dominant disorder, aggregates of mutant huntingtin (Htt) protein are present both in the cytosol and the nucleus^2,3^. Htt is a large, 3144 amino acid protein that has an N-terminal polyglutamine (polyQ) repeat, several HEAT (huntingtin, elongation factor 3, protein phosphatase 2A, TOR1) repeats, a nuclear localization signal, and a C-terminal nuclear export signal^4^. The disease-causing mutation is an expansion of the polymorphic CAG repeat in the first exon of the *huntingtin* (*HTT,* OMIM: 613004) gene that translates to an elongated polyQ repeat in the mutant Htt protein (mHtt)^5^. Repeats of 36 or more glutamines (Qs) are pathogenic, and alleles with ≥ 40 Qs have complete penetrance. mHtt is cleaved by several proteases, including caspases and calpains, that result in the formation of short, toxic, aggregation-prone, polyQ-containing N-terminal mHtt fragments^2^. mHtt induces a complex pathogenesis that negatively affects several key cellular processes, including transcriptional regulation and the ubiquitin–proteasome system (UPS)^6–8^. A plethora of studies indicate the involvement of the UPS in mHtt-induced pathological processes^6^, although several aspects of perturbed UPS function, i.e., the role of deubiqutinatase (DUB) enzymes in HD pathology, are still not clearly elucidated.

Ubiquitin (Ub), a 76 amino acid protein, is encoded by multigene families both in fruit flies and mammals. Members of these gene families encode ubiquitin either in the form of tandem polyubiquitin precursors or fused to ribosomal protein precursors, and the formation of Ub monomers require the activity of DUB enzymes^9–12^. Ubiquitin can be covalently conjugated to target proteins by a tightly controlled, multistep enzymatic mechanism^12^. Monoubiquitination or multiubiquitination of proteins affects their activity and interactions, while polyubiquitination (formation of a ubiquitin chain of at least four units) through the K48 lysine residue marks proteins for proteasomal degradation^13^. Conjugation of Ub to target proteins needs the activity of ubiquitin-activating (E1), ubiquitin-conjugating (E2), and ubiquitin ligase (E3) enzymes, while Ub is released from proteins by hydrolysis via the activity of DUB enzymes^12^. The availability of free ubiquitin monomers is essential for the proper regulation of Ub-dependent processes. The level of monoubiquitin is heavily influenced by DUB enzymes that are required for both the synthesis of Ub and its release from protein substrates^12^. In humans, approximately 100 DUBs were identified to date that belong to six structurally distinct protein families, including the ovarian tumor proteases (OTU), the ubiquitin-specific protease (USP), the ubiquitin C-terminal hydrolases (UCH), the Josephin / Machado-Joseph Deubiquitinases (MJD), and the motif interacting with ubiquitin-containing novel DUB family (MINDY) cysteine protease families, and the Zn-dependent JAMM metalloprotease protein family^14^. Six of these enzymes were shown previously to interact with the Htt protein and/or modulate some aspect of HD pathology^15^.

In this study, we performed a genetic interaction screen in a *Drosophila* model of HD to identify DUBs that have modulatory effects on mHtt-induced pathology. In the fruit fly genome, there are approximately 45 genes encoding DUB enzymes^12^. We tested the effects of altering the activity of 32 genes, encoding DUBs belonging to the OTU, MJD, USP, UCH, or JAMM families, on mHtt-induced phenotypes and found that increased activity of the *Yod1* (*CG4603*) gene, encoding the *Drosophila* ortholog of the human YOD1 protein, suppressed all tested phenotypes in the HD model. We found that *Yod1* overexpression had only minor effects on mHtt aggregation but led to the restoration of the transcriptional activity of the majority of genes dysregulated in the HD model.

## Results

### Genetic screen of deubiquitinases identifies *Yod1* as a modulator of mutant Huntingtin induced pathology

To evaluate the effects of DUB mutants on HD pathology we performed genetic interaction tests by generating flies expressing exon1 of human *Huntingtin* with a 120 residue long polyQ repeat (*Httex1.Q120*)^16^ in the nervous system under the influence of the *elav-GAL* pan-neuronal driver (HD flies) and simultaneously being heterozygous for DUB mutations, transgenes or RNAi constructs. We tested the effects of altered levels of 32 deubiquitinase genes, including six genes of the OTU family (*CG3251*, *CG4968, Deubiquitinating enzyme A* (*Duba*), *ovarian tumor* (*out*), *trabid* (*trbd*), and *Yod1*), one gene of the MJD family (*CG3781*), 18 genes of the USP family (*Cylindromatosis* (*CYLD*), *Deubiquitinating apoptotic inhibitor* (*DUBAI*), *fat facets* (*faf*), *non-stop* (*not*), *puffyeye* (*puf*), *Ubiquitin specific protease 1 (Usp1*), *Usp2*, *Usp5*, *Usp7*, *Usp8*, *Usp10*, *Usp-12-46*, *Usp14*, *Usp15-31*, *Usp20-33*, *Usp30*, *Usp32*, and *Usp47*), two genes of the UCH family (*Ubiquitin carboxy-terminal hydrolase* (*Uch*), and *Uch-L5*), and five genes of the JAMM metalloproteinase family (*CG2224*, *COP9 signalosome subunit 5* (*CSN5*), *CSN6*, *Regulatory particle non-ATPase 8* (*Rpn8*), and *Rpn11*).

**Figure 1:**
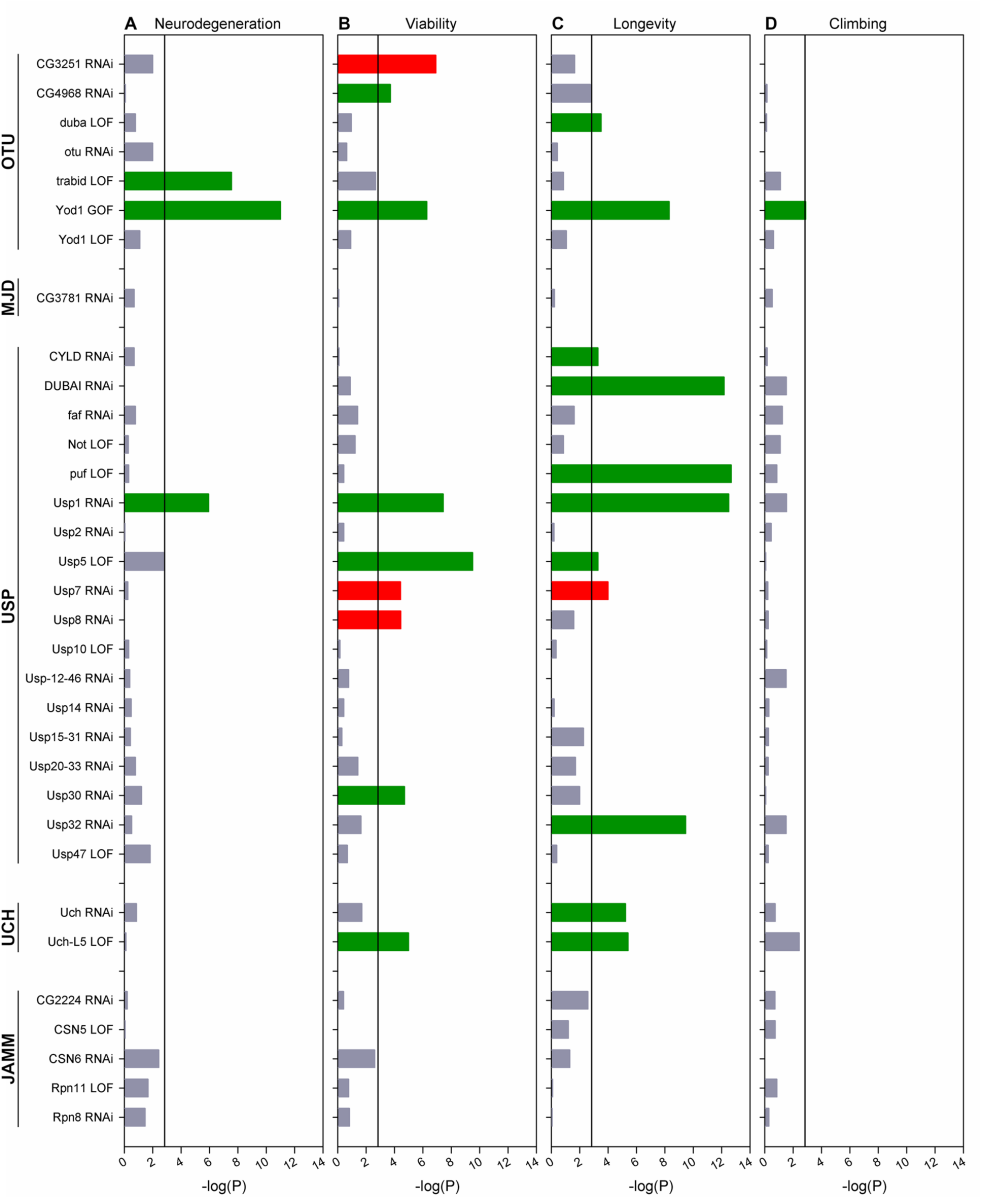
Characterization the effects of DUB mutants on HD phenotypes in genetic interaction crosses. Female flies overexpressing mHtt in the nervous system and simultaneously carrying a genetic element affecting a specific DUB (heterozygous for a loss-of function DUB allele (LOF), or overexpressing a DUB transgene (GOF), or expressing an RNAi construct (RNAi)) were compared to mHtt expressing control female siblings. (**A**) Neurodegeneration was measured as loss of rhabdomeres in the compound eye. (**B**) Viability was determined by comparing the number of emerging mHtt, mHtt-DUB and non-expressing control flies. (**C**) Longevity was analyzed by comparing median lifespans. (**D**) Climbing activity was determined by measuring the vertical distance climbed in 5 s. The X scale shows the − log(P) values of statistical tests (neurodegeneration: t-test, viability: Χ^2^-test, longevity: Fisher’s exact test, climbing: Wilcoxon Sum of Ranks test). Green color indicates significant suppression, red color indicates significant enhancement of phenotypes, and grey color indicates a non-significant effect. The significance threshold line is set at Bonferroni corrected α < 0.05 level for multiple comparison testing.

Neuronal expression of *Httex1.Q120* leads to neuronal degeneration, decreased viability and longevity, and impaired motor activity^16,17^. We investigated the effects of DUB mutants on these four mHtt-induced phenotypes (Fig. 1, Supplementary file S1). The effect on neurodegeneration was analyzed by determining the degeneration of photoreceptor neurons in the compound eye of seven-day-old females by the pseudopupil assay^18^. In *wild-type* flies seven visible rhabdomeres, light gathering structures of photoreceptor neurons, can be visualized in each ommatidium. In *Httex1.Q120* expressing flies neurodegeneration leads to the reduction of the number of intact rhabdomeres^16^. We found that overexpression of *Yod1* (P = 9.7 × 10^–12^, Supplementary Fig. S1) or reduction of *trbd* (P = 2.7 × 10^–8^) or *Usp1* (P = 1.13 × 10^–6^) significantly ameliorated mHtt-induced neurodegeneration in the eye (Fig. 1A).

Neuronal expression of *Httex1.Q120* causes a marked reduction of viability measured as the number of eclosing HD flies compared to non-HD control siblings^16^. By analyzing the effects of DUB mutants on mHtt-induced reduced viability we found that overexpression of *Yod1* (P = 5.29 × 10^–7^) or reduction of *CG4968* (P = 1.9 × 10^–4^), *Usp1* (P = 3.7 × 10^–8^), *Usp5* (P = 3.12 × 10^–10^), *Usp30* (P = 1.98 × 10^–5^), or *Uch-L5* (P = 1.02 × 10^–5^) increased viability while reduction of *CG3251* (P = 1.2 × 10^–7^), *Usp7* (P = 3.7 × 10^–5^) or *Usp8* (P = 3.7 × 10^–5^) reduced viability (Fig. 1B).

Expression of *Httex1.Q120* significantly reduces the lifespan of flies^16,17^. By longevity analysis we found that overexpression of *Yod1* (P = 7 × 10^–4^) or reduction of *duba* (P = 3 × 10^–4^), *CYLD* (P = 5 × 10^–4^), *DUBAI* (P = 6.4 × 10^–13^), *puf* (P = 2 × 10^–13^), *Usp1* (3 × 10^–13^), *Usp5* (P = 5 × 10^–4^), *Usp32* (P = 3.4 × 10^−10^), *Uch* (P = 5.9 × 10^–6^) or *Uch-L5* (P = 3.8 × 10^–6^) increased while reduction of *Usp7* (P = 1 × 10^–4^) decreased the median lifespan of HD flies (Fig. 1C).

Finally, we investigated the effects of DUB mutants on the impaired motor activity of HD flies^16^ by climbing assays and found that the climbing activity of HD flies overexpressing *Yod1* was significantly increased (P = 0.0013) (Fig. 1D).

Thus, in our screen genetic manipulation of two DUB genes, *Yod1* and *Usp1*, had significant effects on at least three of the analyzed disease phenotypes and we considered these HD modifier candidates. *Yod1* encodes for the dYOD1 ubiquitinyl hydrolase enzyme whose closest human ortholog is the YOD1 deubiquitinase (sequence identity: 42%, similarity: 58%). The *Yod1*^*EY07831*^ allele that suppressed HD induced pathology carries a P{EPgy2} element in the 5’-UTR region of the gene that drives the over-expression of *Yod1* in the presence of a GAL4 driver. *Usp1* encodes for a deubiquitinase whose closest human ortholog is the ubiquitin specific peptidase 1 protein (USP1, sequence identity: 24%, similarity: 38%). The *Usp1*^*JF02992*^ transgenic strain used in the tests carries a P{TRiP.JF02992} transgene that provides a GAL4 dependent down-regulation of *Usp1* by RNAi.

**Figure 2:**
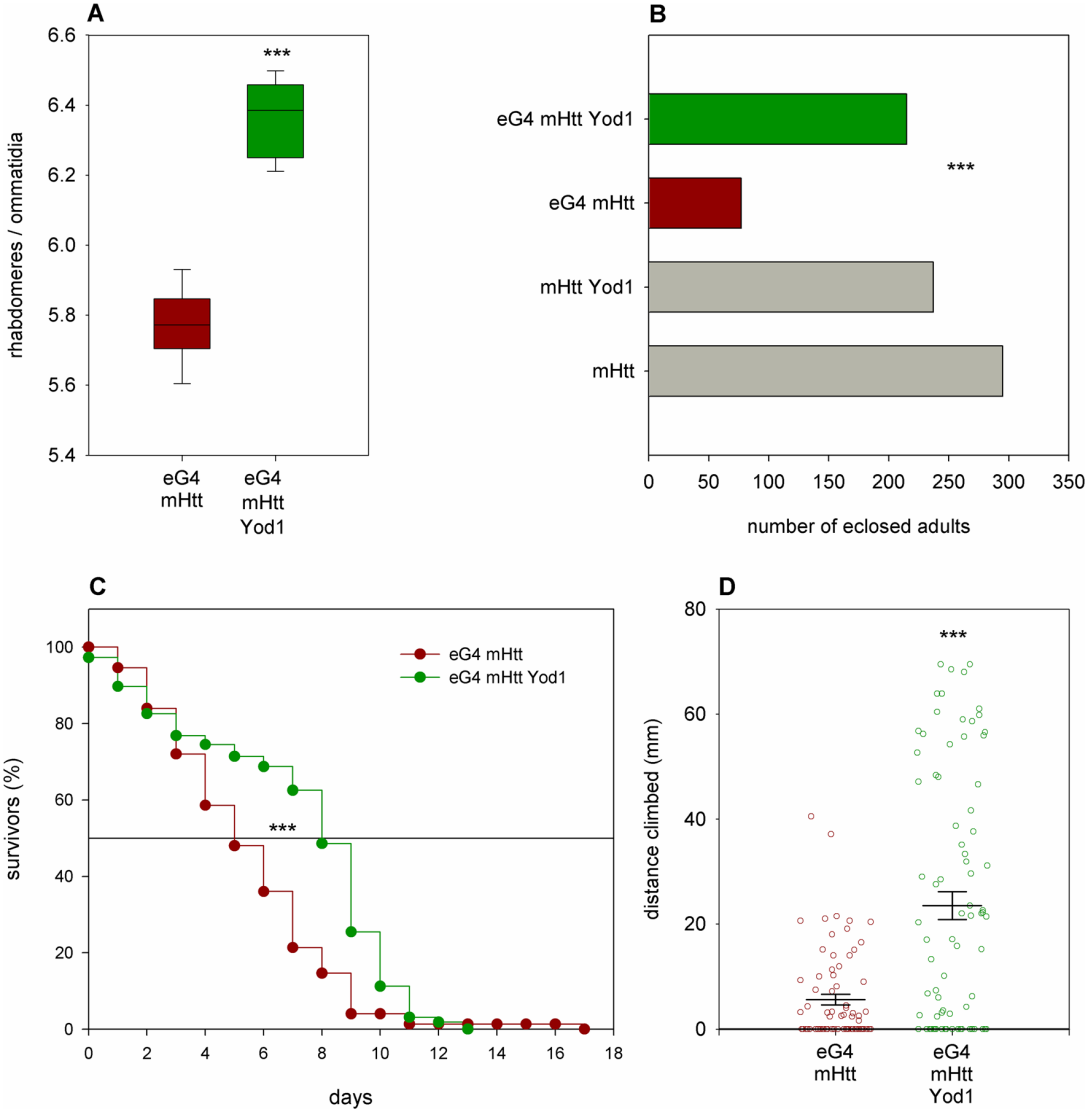
Overexpression of *Yod1* rescues mHtt-induced phenotypes. (**A**) Neuronal (*elav-GAL4* (eG4) driven) overexpression of the *Yod1.flag* transgene significantly reduces degeneration of photoreceptor neurons (P = 3.59 × 10^–10^, n = 10/10). Boxes show 25th, 50th and 75th quartiles of the average number of rhabdomeres per ommatidia in compound eyes, whiskers represent 10th and 90th quartile values. (**B**) Flies expressing mHtt under the influence of the neuronal *elav-GAL4* driver have lower viability than non-expressing control siblings (gray). Simultaneous overexpression of *Yod1* improves viability of HD flies close to wild-type levels (P = 1.02 × 10^–15^, n = 824). (**C**) Overexpression of *Yod1* significantly increases the median lifespan of mHtt expressing flies (P = 8.9 × 10^–13^, n_exp_ = 224, n_cont_ = 75). (**D**) Overexpression of *Yod1* significantly ameliorates motor dysfunction of HD flies (P = 4.08 × 10^–6^, n_exp_ = 83, n_con_ = 75). Datapoints show vertical distances climbed by individual flies in 5 s, horizontal lines and error bars indicate population mean values and the standard error of mean, respectively. *** indicates P < 0.001.

To validate our findings with *Yod1* and *Usp1* we tested independent lines with similar functional effects on gene activity. In the case of *Yod1* we generated a transgenic *UAS-Yod1.flag* strain by φC31 integrase mediated site-specific integration of a pTWF.attB-Yod1 construct in the third chromosomal *ZH-86Fb* docking site and tested the effects of this element on mHtt-induced phenotypes. Over-expression of *UAS-Yod1.flag* transgene significantly ameliorated all analyzed phenotypes. In the eye the average number of rhabdomeres per ommatidia in flies co-expressing *Httex1.Q120* and *Yod1* increased to 6.36 ± 0.03 (average ± SEM) from 5.77 ± 0.03 in *Httex1.Q120* expressing controls (P = 3.59 × 10^–10^, n = 10/10, Fig. 2A). Relative viability increased 3.5-fold from 26.1% in *Httex1.Q120* expressing controls to 90.7% in flies co-expressing *Httex1.Q120* and *Yod1* (P = 1.02 × 10^–15^, n = 824, Fig. 2B) and median lifespan increased to 9.94 days from 5.81 days in controls (P = 8.9 × 10^–13^, n of the experimental category (n_exp_) = 224, n of the control category (n_cont_) = 75, Fig. 2C). Motor activity was also significantly increased (P = 4.08 × 10^–6^, Fig. 2D) in *Yod1* overexpressing HD flies as they climbed a larger distance vertically in 5 s (median: 17.1 mm, mean 23.5 mm, n = 83) than *Httex1.Q120* expressing controls (median: 0 mm, mean: 5.6 mm, n = 75). We also tested the effects of Yod1 in flies expressing a non-pathological *HTT* transgene, *Httex1.Q25*, that produces a similar protein as *Httex1.Q120* but with a 25 residues long polyQ repeat. Neither flies expressing *Httex1.Q25* alone, nor ones co-expressing *Httex1.Q25* and *Yod1* showed signs of retinal neurodegeneration (Supplementary Fig. S2A) or reduced viability (Supplementary Fig. S2B). Flies co-expressing *Yod1* and *Httex1.Q25* climbed a significantly (P = 0.008) larger vertical distance in 4 s (median: 37.8 mm, mean: 37 mm, n = 50) than their *Httex1.Q25* expressing siblings (median: 28 mm, mean: 26.4 mm, n = 50) (Supplementary Fig. S2C). Thus, overexpression of *Yod1* increased climbing speed in “healthy” flies, although the scale of this increase was not as large either in absolute or relative terms as in HD flies.

In the case of *Usp1* we used a PBac{PB} transposon induced loss-of-function allele, *Usp1*^*c05664*^, for validation and found that this allele had minor and conflicting effects: it improved the number of eclosed flies but reduced their climbing ability, and did not have a significant modulatory effect on either neurodegeneration or longevity (Supplementary Fig. S3).

### *Yod1* has mixed effects in a genetic Alzheimer’s disease model

**Figure 3:**
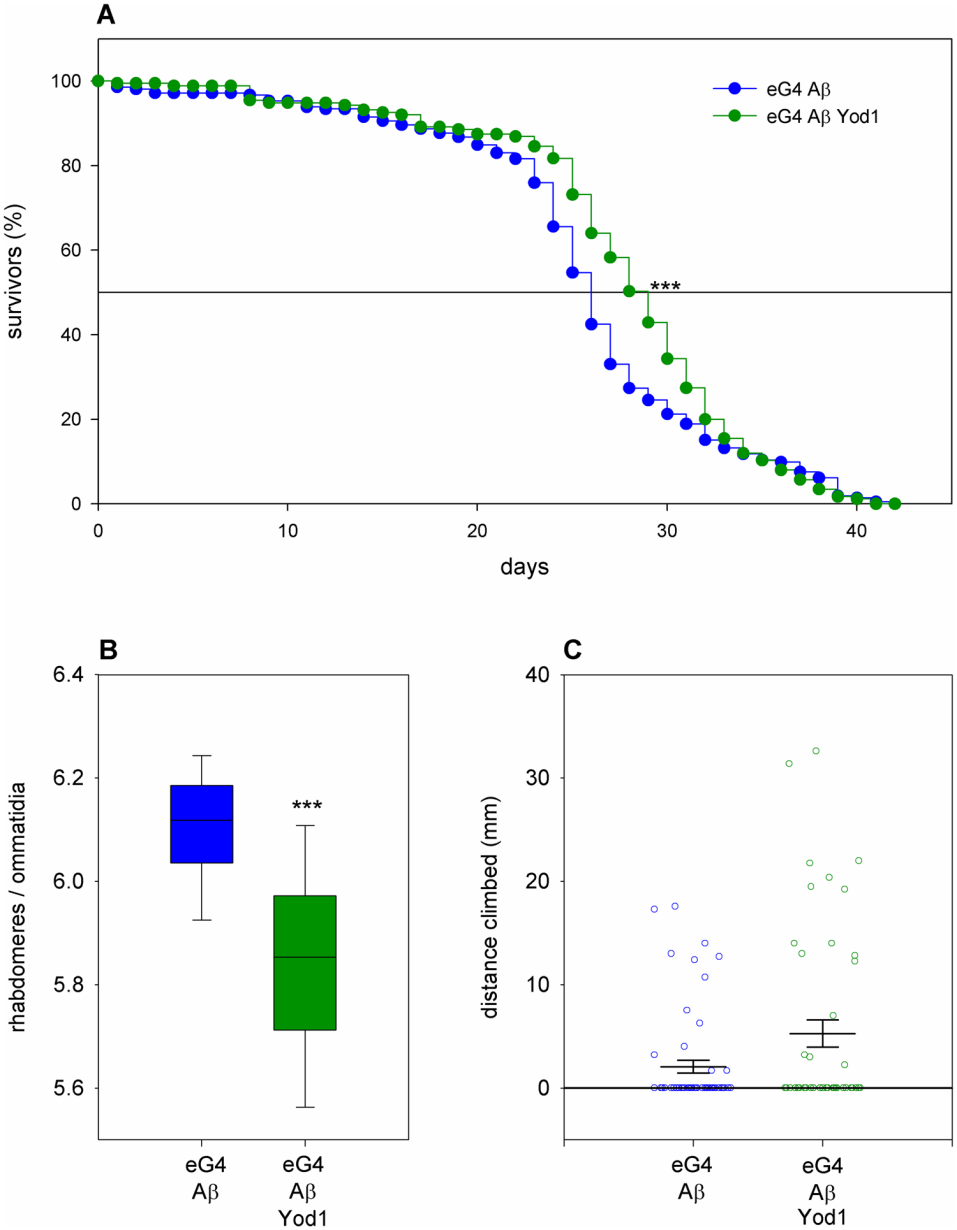
Overexpression of *Yod1* has mixed effects on Aβ induced phenotypes. (**A**) Simultaneous neuronal overexpression of the *Yod1.flag* transgene significantly increases the median lifespan of Aβ expressing flies (P = 2.8 × 10^–8^, n_exp_ = 175, n_cont_ = 212), (**B**) but it also significantly increases the degeneration of photoreceptor neurons (P = 5.8 × 10^–4^, n = 10/10). Boxes show 25th, 50th and 75th quartiles of the average number of rhabdomeres per ommatidia in compound eyes, whiskers indicate 10th and 90th quartile values. (**C**) Overexpression of *Yod1.flag* does not have a significant effect on the climbing ability of Aβ expressing flies. Data points show vertical distances climbed by individual flies in 5 s, horizontal lines and error bars indicate population mean values and the standard error of mean, respectively. *** indicates P < 0.001.

We were curious to find out whether the modulatory effects of *Yod1* were specific for mutant Htt induced pathology or similar effects could be observed in other protein misfolding disease models. Therefore, we tested the effects of *Yod1* overexpression in an Alzheimer’s disease (AD) model, which is based on the expression of human amyloid beta peptide (Aβ, amino acids 1–42)^19^. Neuronal expression of *UAS-Aβ* transgene does not reduce the number of eclosed adults, but shortens lifespan, impairs motor activity^19^, and causes neurodegeneration. We tested the effects of *Yod1* co-expression on the latter three phenotypes using the *Yod1.flag* transgene and found that although it increased the lifespan of Aβ expressing flies (median lifespan increased to 29.4 days from 26.47 days in controls, P = 2.8 × 10^–8^, n_exp_ = 175, n_cont_ = 212, Fig. 3A), it significantly enhanced neurodegeneration (P = 5.8 × 10^–4^, n = 10/10) in the eyes of 10-day-old flies to 5.84 ± 0.06 (average ± SEM) from 6.11 ± 0.03 in controls (Fig. 3B). Its effect on the motor activity of 10-day-old AD flies was not significant (P = 0.085, n_exp_ = 59, n_cont_ = 47, Fig. 3C).

We repeated these experiments using the *Yod1*^*EY07831*^ overexpression allele and got similar results: significantly improved lifespan but reduced neuronal survival with no significant change in climbing speed (Supplementary Fig. S4). As the all-round phenotypic improvements that we observed in the HD model upon *Yod1* overexpression were not characteristic for the AD model we concluded that the effects of *Yod1* are rather specific to mHtt-induced pathology than having a generic positive effect in response to accumulation of misfolded proteins.

### *Yod1* overexpression moderately affects the size distribution of mHtt aggregates

**Figure 4:**
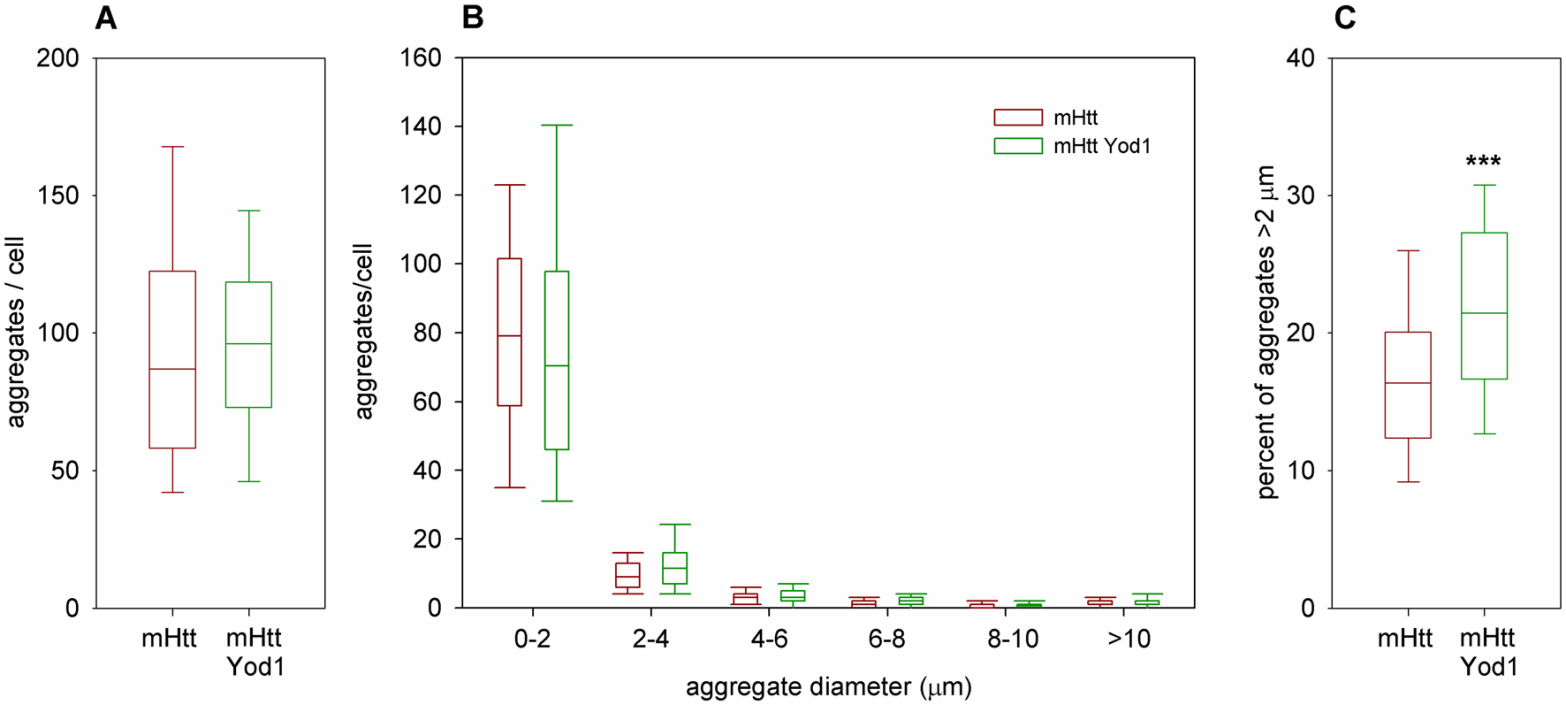
The effect of *Yod1* overexpression on mHtt aggregation. (**A**) Overexpression of *Yod1* does not alter the total number of mHtt aggregates (P = 0.48, n_exp_ = 96, n_cont_ = 74). (**B**) The distribution of the number of mHtt aggregates of different apparent diameters in flies expressing *HTT.96Q.Cerulean* alone or co-expressing *HTT.96Q.Cerulean* and *Yod1*. (**C**) *Yod1* overexpression moderately increases the proportion of larger (> 2 μm) aggregates (P = 5 × 10^–6^, Wilcoxon Sum of Ranks test). The effect on the aggregation of HTT.96Q.Cerulean protein was analyzed in larval salivary gland cells via confocal microscopy. Boxes show 25th, 50th and 75th quartiles, whiskers mark 10th and 90th quartile values. *** indicates P < 0.001.

mHtt is an aggregation prone protein and disease modifying mechanisms might lead to alteration in the aggregation properties of mHtt. To investigate whether the positive effects of *Yod1* overexpression on disease pathology concur with changes in mHtt aggregation we expressed a *UAS-HTT.96Q.Cerulean* transgene, encoding the first exon of human HTT with 96 residue long polyQ region fused with a Cerulean fluorescent protein, either alone or simultaneously with *Yod1*^*EY07831*^ and analyzed the number and size of mHtt aggregates in salivary gland cells via confocal microscopy (Fig. 4, Supplementary Fig. S5). We found no significant difference in the average number of visible aggregates per cell (100 ± 6 (average ± SEM), n = 96 in flies co-expressing mHtt and *Yod1*, and 96.1 ± 3.6, n = 74 in mHtt expressing controls, P = 0.48) (Fig. 4A). However, we could observe a mild but statistically significant shift towards larger aggregates as the ratio of aggregates with apparent size of > 2 µm increased to 22.1 ± 0.8% from 17 ± 0.8% in controls (P = 5 × 10^–6^, Wilcoxon Sum of Ranks test) (Fig. 4B,C).

### Yod1 overexpression results in partial restoration of the transcriptional program in HD flies

To identify molecular pathways that are modulated in mHtt expressing flies upon *Yod1* overexpression we performed transcriptome analysis. We applied RNA-sequencing on head samples of 5-day-old female flies carrying the *elav-GAL4* driver alone (control), or overexpressing *Httex1.Q120*, *Yod1*, or *Httex1.Q120* and *Yod1* simultaneously under the influence of *elav-GAL4*. The data validated significant overexpression of *Yod1* in *elav-GAL4/*+*; Yod1*^*EY07831*^*/*+ and *elav-GAL4/*+ *; Httex1.Q120/*+*; Yod1*^*EY07831*^*/*+ flies (Supplementary Fig. S6A) and also proved that the expression level of mutant Huntingtin is not reduced in *elav-GAL4/*+*; Httex1.Q120/*+*; Yod1*^*EY07831*^*/*+ flies (Supplementary Fig. S6B), implying that the phenotypic effects observed in genetic interaction tests are not due to the downregulation of the *Httex1.Q120* transgene.

**Figure 5:**
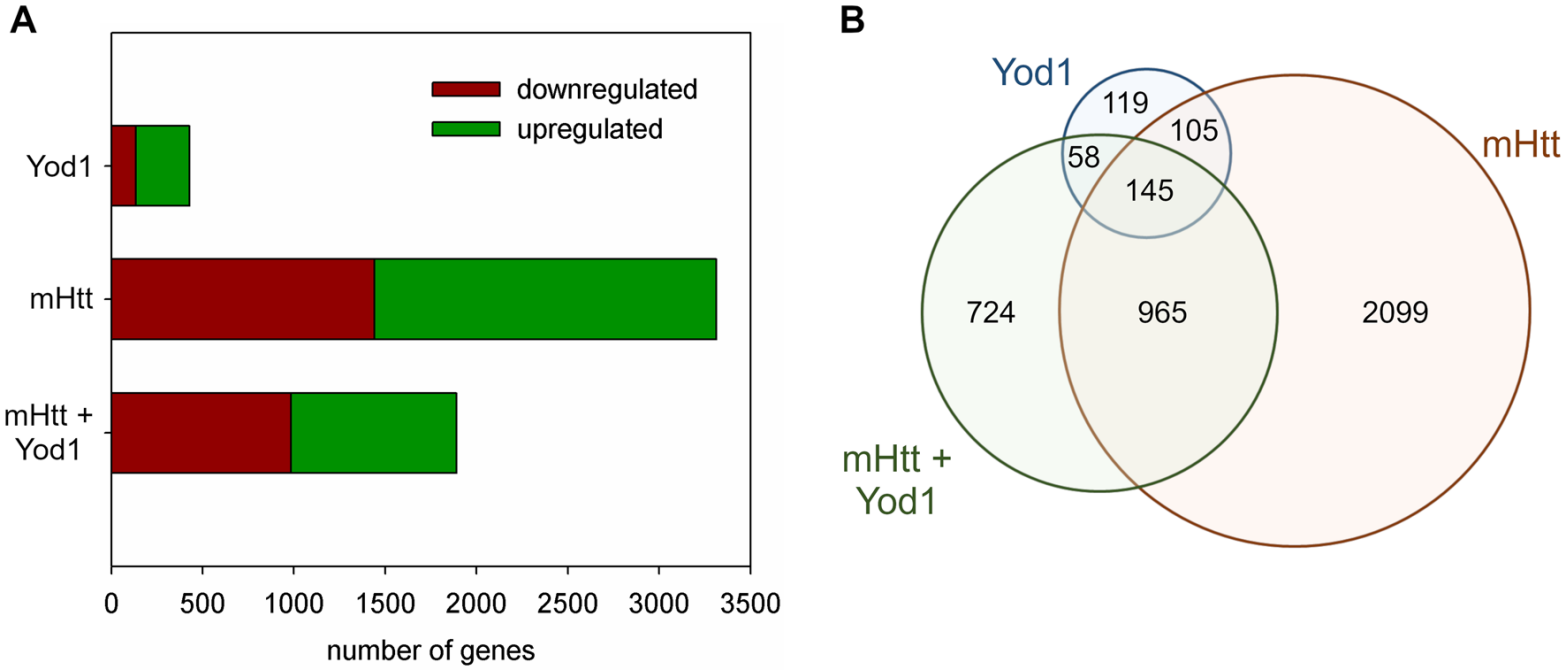
RNA-seq analysis indicates partial restoration of transcriptional dysregulation upon *Yod1* overexpression in HD flies. (**A**) While overexpression of *Yod1* have moderate transcriptional effects, overexpression of *mHtt* leads to the dysregulation of 3314 genes. Overexpression of *Yod1* in HD flies reduces the number of dysregulated genes by 43%. The graph shows the number of genes showing altered expression compared to *elav-GAL4* control at significance level α < 0.05. (**B**) Venn diagrams show the number of genes having significantly altered transcript levels compared to the *elav-GAL4* control in the three experimental genotypes.

*Yod1* overexpression had relatively mild effects on transcription. It led to altered expression of 427 genes compared to the *elav-GAL4* control, out of which 135 genes were downregulated and 292 genes were upregulated at adjusted significance level of α = 0.05 (Fig. 5A, Supplementary file S2). *Httex1.Q120* expressing HD flies showed a more profound transcriptomic response: 1441 genes were downregulated while 1873 were upregulated, altogether 3314 genes had altered expression levels (Fig. 5A, Supplementary file S2). In flies co-expressing *Httex1.Q120* and *Yod1* 985 genes were downregulated and 907 were upregulated (1892 genes total) compared to the *elav-GAL4* control (Fig. 5A, Supplementary file S2). Thus, overexpression of *Yod1* led to a 43% reduction in the number of dysregulated genes in HD flies. Furthermore, we found that a surprisingly large set of 250 genes showed altered transcript levels in both *Yod1* and *Httex1.Q120* overexpressing flies (Fig. 5B). The considerable overlap between the mHtt and Yod1 sets suggests that Yod1 overexpression modulates molecular processes that are also affected by the presence of mHtt.

We performed Gene Set Enrichment Analysis (GSEA) and found that the most significantly affected biological processes by *Yod1* overexpression are related to protein biosynthesis and folding, including Gene Ontology Biological Process (GO BP) terms cytoplasmic translation (GO:0002181, P = 5.58 × 10^–44^), ribosome assembly (GO:0042255, P = 9.43 × 10^–7^), 'de novo' posttranslational protein folding (GO:0051084, P = 4.37 × 10^–5^), ribonucleoprotein complex assembly (GO:0022618, P = 4.47 × 10^–4^), cellular response to topologically incorrect protein (GO:0035967, P = 0.0012), and response to unfolded protein (GO:0006986, P = 0.0012), among others (Supplementary file S3). GSEA also indicated that the transcriptional changes observed in *Yod1* overexpressing flies are significantly enriched in datasets of models of two human diseases, HD (P = 0.017) and Machado-Joseph disease (Spinocerebellar Ataxia 3, P = 0.041), another polyQ induced neurodegenerative disorder. This finding, together with the above described significant overlap between *mHtt* and *Yod1* induced transcriptomic changes suggests that overexpression of *Yod1* influences molecular pathways that are important in mHtt-induced pathology.

**Figure 6:**
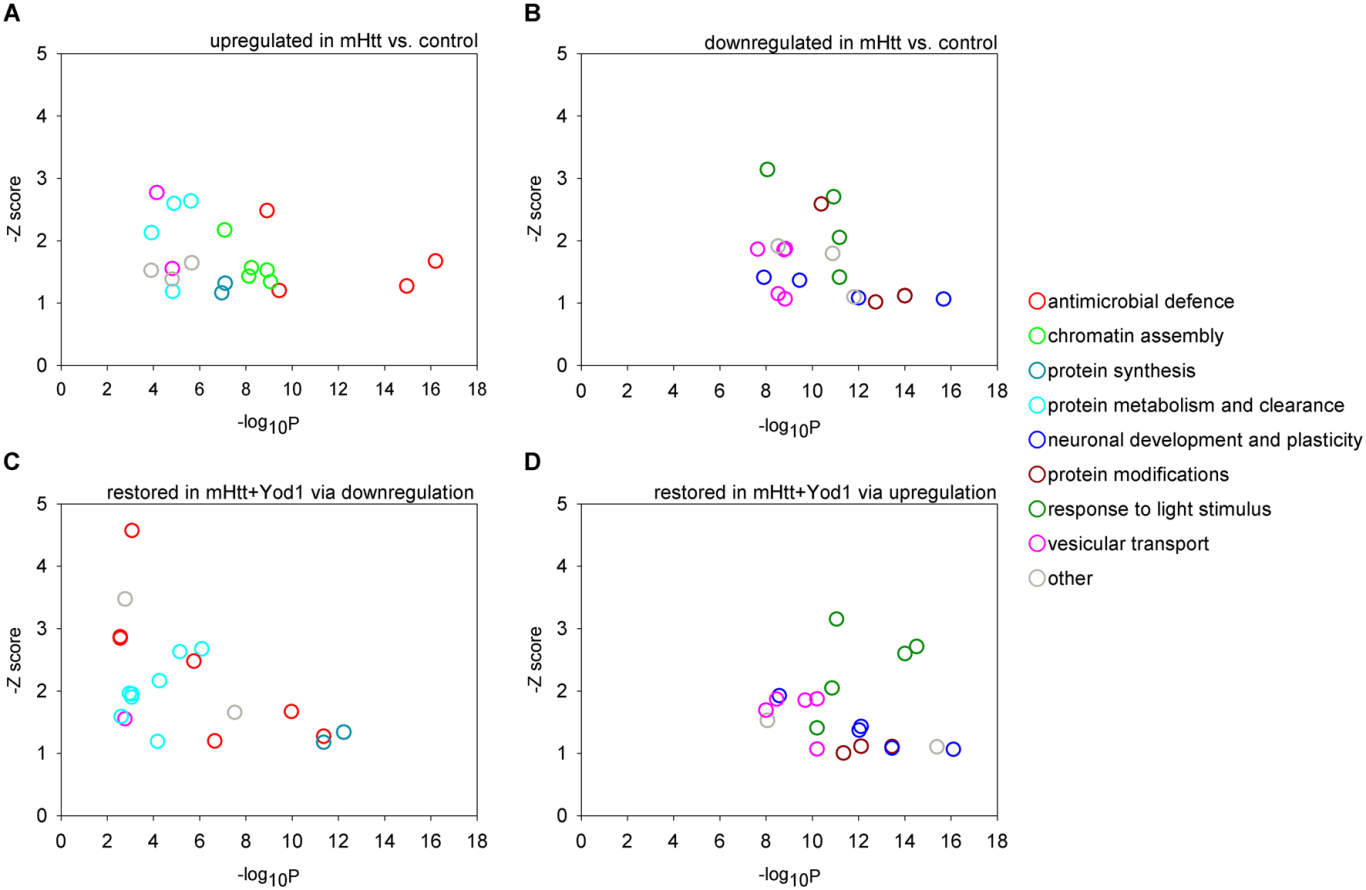
Overexpression of *Yod1* partially restores biological processes affected by mHtt. (**A**) Top 20 most significantly enriched GO biological process terms (colored based on functional categories) of genes upregulated or (**B**) downregulated in head samples of *mHtt* expressing flies vs. non-expressing siblings. (**C**) Top 20 most significantly enriched GO biological process terms of genes whose expression was fully or partially restored by downregulation or (**D**) upregulation in *Yod1* co-expressing HD flies compared to HD flies. Overlaps between functional categories enriched in A/C or B/D indicate that overexpression of Yod1 restores the expression of genes dysregulated as a result of mHtt expression. Gene set enrichment analysis was performed using FlyEnrichr, adjusted P values were calculated by applying Benjamini–Hochberg correction for multiple hypothesis testing, z-score corresponds to the deviation from the expected rank^20^.

The transcriptional changes in *Httex1.Q120* expressing HD flies showed significant enrichment in gene datasets of models of neurodegenerative diseases, such as HD (P = 6.37 × 10^–8^), Parkinson's disease (P = 7.49 × 10^–8^), and tauopathy (P = 1.22 × 10^–6^), among others, validating the HD model (Supplementary file S3). The genes upregulated in HD flies were enriched in GO BP terms related to antimicrobial defense, chromatin assembly, protein synthesis, and protein metabolism and clearance (Fig. 6A, Supplementary file S3). The set of downregulated genes in HD flies were enriched in GO BP terms related to neuronal development and plasticity, protein modifications, response to light stimulus, and vesicular transport (Fig. 6B, Supplementary file S3).

To directly analyze the effects of *Yod1* on mHtt-induced transcriptional alterations we compared RNA-seq data of flies expressing *Httex1.Q120* only with those of flies co-expressing *Httex1.Q120* and *Yod1*. We found that co-expression of *Yod1* in HD flies led to the significant downregulation of 1674 genes, and upregulation of 1381 genes (at α = 0.05) compared to flies expressing *Httex1.Q120* alone (Supplementary file S2). The top five upregulated genes were *Integrator 12* (encodes a protein involved in snRNA transcription), *CG4611* (encodes a predicted tRNA binding protein), *CG18577* (encodes a protein of unknown function), *yellow* (encodes a protein involved in melanization), and *CG31219* (encodes a predicted serine-type endopeptidase); while the top 5 downregulated genes were *hoka* (encodes an endothelial barrier protein), *Lsp1alpha* (encodes a larval storage protein), *Mucin 68Ca* (encodes a mucin protein), *Cecropin C* (encodes an antibacterial protein), and *Induced by Infection* (encodes a micropeptide that responds to wasp infection)^21^. In case of 59.3% of the 3055 differentially expressed genes overexpression of *Yod1* fully or partially restored the level of transcription in HD flies, i.e. we measured upregulation of genes that were downregulated in HD flies compared to *elav-GAL4* control (748 genes), or conversely, we measured downregulation of genes that were upregulated in HD flies compared to *elav-GAL4* control (1065 genes). Not surprisingly, the sets of genes with restored expression showed most significant enrichments of similar GO BP terms that were described earlier as enriched among dysregulated genes in HD flies, i.e. terms related to antimicrobial defense, protein synthesis, and protein metabolism and clearance in the set of genes downregulated upon *Yod1* co-overexpression (Fig. 6C, Supplementary file S3), and terms related to response to light stimulus, neuronal development and plasticity, protein modifications, and vesicular transport in the set of genes upregulated upon *Yod1* co-expression in HD flies (Fig. 6D, Supplementary file S3).

**Figure 7:**
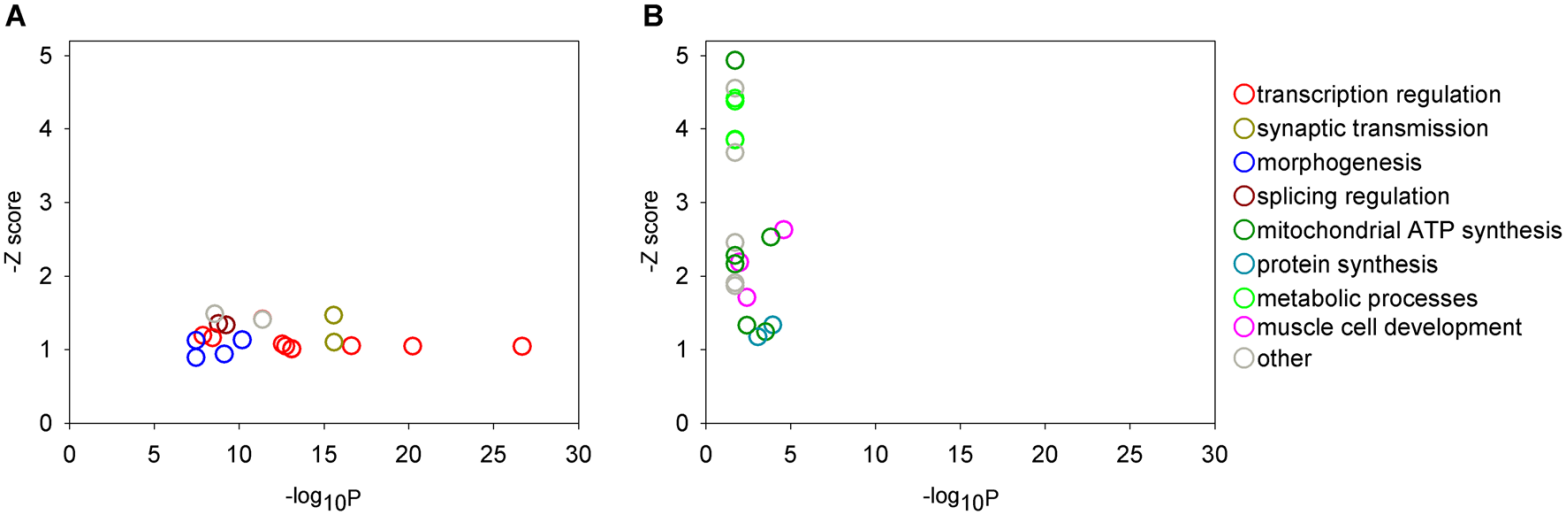
Overexpression of *Yod1* in HD flies influences biological processes that might contribute to the suppression of symptoms. (**A**) Top 20 most significantly enriched GO biological process terms (colored based on functional categories) of genes that are not dysregulated in HD flies but upregulated or (**B**) downregulated in head samples of flies co-expressing *Yod1* and *mHtt* expressing flies vs. HD siblings. Gene set enrichment analysis was performed using FlyEnrichr, adjusted P values were calculated by applying Benjamini–Hochberg correction for multiple hypothesis testing, z-score corresponds to the deviation from the expected rank^20^.

Another interesting group contains sets of those genes, which were not dysregulated in HD flies but show altered expression in *Yod1* co-expressing HD flies compared to HD siblings. Specifically, we identified 615 genes, which were upregulated, and 545 genes, which were down-regulated as a response to *Yod1* overexpression in HD flies but were not dysregulated in *mHtt* expressing flies compared to *elav-GAL4* controls. The upregulated genes are enriched in GO BP categories related to regulation of transcription, mRNA processing, morphogenesis and synaptic transmission (Fig. 7A, Supplementary file S3), while the downregulated genes are enriched in GO BP terms related to protein synthesis, mitochondrial ATP synthesis, metabolic processes, and muscle cell development (Fig. 7B, Supplementary file S3). We hypothesize that altered regulation of these processes might play a role in the suppression of HD pathology and phenotypes upon *Yod1* overexpression.

## Discussion

Accumulation of misfolded proteins is a characteristic feature of numerous neurodegenerative diseases indicating that dysfunction of the cellular protein clearance system is a common theme in these disorders^22^. One of the major protein clearance mechanisms of eukaryotic cells is the ubiquitin–proteasome system that participates in protein quality control by degrading misfolded proteins^23^. Deubiquitinase enzymes are critical components of the UPS as they are required both for the release of Ub monomers during Ub protein synthesis and for the hydrolysis of conjugated Ub from protein substrates^24^. Hence, DUBs make a substantial contribution to the preservation of the monoubiquitin pool, regulating proteolysis and ensuring proper proteostasis. Unsurprisingly, DUB enzymes were implicated in the pathology of various neurodegenerative disorders, including Alzheimer’s disease, Parkinson’s disease (PD), Amyotrophic lateral sclerosis (ALS), and HD^25^. In the case of HD, previous studies indicated the involvement of at least four deubiquitinases in pathology, ataxin-3^26^, YOD1^27^, Usp12^28^, and Usp19^29^.

Here we described a project aimed at the identification of DUBs capable of modulating pathology in an animal model of HD. We tested genetic interactions of a mutant Htt transgene with 32 genes encoding deubiquitinases belonging to five DUB protein families and found that overexpression of *Yod1*, a DUB belonging to the OTU (ovarian tumor) superfamily of cysteine proteases, reduced the degeneration of photoreceptor neurons, and improved motor performance, viability and longevity of HD flies. *Yod1* overexpression did not have a similarly overall positive effect on the phenotypes of Aβ_1-42_ expressing AD flies, suggesting that its effects might be at least in part specific to mHtt induced pathology in the fly model.

Its human ortholog, YOD1 (protein identity: 42%, similarity: 58%), is an enzymatically active cysteine protease with K11-, K48- and K63-linked ubiquitin-specific deubiquitinase activity^30,31^. YOD1 participates in several biological processes that can be linked to protein misfolding induced neurodegeneration, including ER-associated degradation (ERAD), autophagic clearance of damaged lysosomes (Endo-Lysosomal Damage Response, ELDR), immunomodulation, and regulation of the Hippo and NF-κB signaling pathways^30,32–35^. The most-well characterized role of YOD1 is the one it plays in ERAD. Several lines of evidence indicate that YOD1 plays a role in ERAD dependent degradation of both ubiquitinated and non-ubiquitinated proteins^30,31,36,37^. Depending on the protein substrate YOD1 is involved in (at least) two distinct steps of ERAD: retro-translocation of the misfolded protein to the cytosol and subsequent proteasomal degradation^36^. One of the main interacting partners of YOD1 in ERAD related functions is ATPase p97/Valosin-containing protein (VCP) that participates in the retro-translocation of misfolded proteins from the ER to the cytosol. In case of ubiquitinated proteins YOD1 is required for the deubiquitination of Ub-conjugated ERAD substrates and reduced YOD1 activity results in the accumulation of Ub-conjugated forms of these proteins through disturbing p97-associated functions^30,37^. In contrast, the effect of YOD1 on non-ubiquitinated protein substrates, such as cholera toxin A1 (CTA1) and nonglycosylated yeast pro-α factor, is dissimilar as it antagonizes their retro-translocation via a mechanism not involving substrate ubiquitination^31^. This suggests that this effect might be a consequence of regulating the ubiquitination state of the components of the ERAD machinery. In the case of proteins that are dislocated from the ER in a p97 and YOD1 independent manner, YOD1 with p97 was shown to be required for cytosolic deglycosylation and targeting to the proteasome^36^.

YOD1 was shown previously to be upregulated upon various stress conditions in vitro, including inhibition of proteasome, ER stress, uncoupling of mitochondrial oxidative phosphorylation, and by the expression of mutant Htt (HttQ74)^27^. Excess YOD1 reduced the level of HttQ74 protein in a cell culture model of HD, and also attenuated HttQ74, α-synuclein, and synphilin-1 induced decreased cell viability in a deubiquitinase activity dependent manner^27^. In human brain samples YOD1 was shown to co-localize with α-synuclein-positive Lewy bodies in PD patients but it did not co-localize with senile plaques and neurofibrillary tangles in AD patients, nor with neuronal nuclear inclusions in HD patients^27^, suggesting that in the latter disorders YOD1 does not exert its effects on the aggregated forms of disease related proteins. Our aggregate analysis corroborates these observations as we could detect only a very mild effect on mHtt aggeragates in *Yod1* overexpressing flies.

To shed light on the biological processes that are adjusted along with the Yod1-dependent suppression of HD pathology, we applied RNA-seq analysis. Neuronal overexpression of *Yod1* even in wild-type flies provided clues suggesting that it might modulate molecular processes important in HD pathology. By performing GSEA we found that differentially expressed genes (DEGs) in *Yod1* overexpressing flies are enriched in protein folding related GO BP terms that are also enriched in human presymptomatic HD striatal and cortical tissues, including 'de novo' posttranslational protein folding, chaperone-mediated protein folding, and response to unfolded protein^8^. GSEA also showed that DEGs in *Yod1* overexpressing flies are significantly enriched in datasets of models of only two human disorders, Huntington’s disease and Machado-Joseph disease (Spinocerebellar Ataxia Type 3), both of which are polyglutamine-induced neurodegenerative disorders^38^. Furthermore, by comparing our RNA-seq datasets we also found that DEGs in HD flies were enriched among DEGs in *Yod1* overexpressing flies.

In HD flies, *Yod1* overexpression reduced the number of DEGs by 43% and fully or partially restored the expression of a number of genes involved in biological processes that are also significantly affected in human HD brain samples. Thus, it led to a substantial improvement of mHtt-induced dysregulation of gene activity indicating a profound cellular effect. Interestingly, DEGs whose expression was normalized by upregulation are enriched in several GO BP categories that also show enrichment in late striatal HD patient samples (BP terms related to axonal guidance, neuronal differentiation, synaptic transmission), while DEGs whose expression was normalized by downregulation are enriched in several GO BP categories that show enrichment in presymptomatic HD patient samples (BP terms related to immune effector, and apoptotic processes)^8^. These findings indicate, that increased *Yod1* activity results in fundamental changes in cellular physiology that influence not only protein homeostasis but also the transcriptional program, thereby having a profound positive impact on mHtt-induced pathology.

However, as the activities of Yod1 are not directly related to transcription, we believe that its effect on transcript levels might be indirect. Ubiquitylation/deubiquitylation regulates the stability and/or activity of a variety of factors directly regulating transcription^39^, therefore figuring out which of these factors and/or processes have significant importance in Yod1-dependent rescue of HD pathology will require further studies. Some promising leads for these inquiries can be the Hippo pathway and chromatin structure regulation by histone methylation. Based on KEGG pathway analysis components of the Hippo signaling pathway, which is dysregulated in HD^40^ and regulated by YOD1^35^, are significantly enriched (P_adj._ = 0.0071) among genes differentially expressed in HD + Yod1 vs. HD flies (these genes are: *Act87E*, *Act42A*, *aPKC*, *app*, *baz*, *bsk*, *dlg1*, *crb*, *dco*, *fred*, *Gug*, *l(2)gl*, *lft*, *Patj*, *sd*, *sdt*, *tws*, *wgn*, and *14-3-3zeta*). Another possible lead is related to the regulation of chromatin structure. Based on GSEA analysis using Flyenrichr with the “Transcription Factors from DroID 2015” dataset genes that are regulated by the histone H3 lysine K4 specific, activating histone methyltransferase trx are enriched (P_adj._ = 1.037 × 10^–140^), while genes that are regulated by the histone H3 lysine K27 specific, repressing histone methyltransferase E(z) are depleted (P_adj._ = 3.617 × 10^–34^) among genes differentially expressed in HD + Yod1 vs. HD flies. In one of our previous studies, we showed that the availability of these methyltransferase enzymes influences HD pathology^41^. Thus, we find it conceivable that Yod1 exerts its pathology-modifying effects, at least in part, by influencing the stability or activity of proteins and macromolecular complexes directly regulating chromatin structure and/or transcription.

## Materials and methods

### *Drosophila* stocks and husbandry

*Drosophila* stocks were maintained on standard *Drosophila* medium (3% dry yeast, 4% cornmeal, 2% wheat flour, 9% glucose, 0.7% agar, 0.15% Tegosept) at 18 °C and expanded at 25 °C. Crosses were made at 25 °C unless noted otherwise.

The *w*; *UAS-Httex1.Q120* and *w*; *UAS-Httex1.Q25* stocks^16^ were kind gifts of J. Lawrence Marsh (University of California, Irvine, CA, USA).

The following stocks: *w*^*1118*^; *P{w*^+*mGT*^ = *GT1}Rpn11*^*BG01694*^/CyO, *w*^*1118*^; *PBac{w*^+*mC*^ = *PB}Usp1*^*c05664*^, *y*^*1*^* v*^*1*^; *P{y*^+*t7.7*^* v*^+*t1.8*^ = *TRiP.JF02992}attP2*, *y*^*1*^* w*^*67c23*^; *P{y*^+*mDint2*^* w*^+*mC*^ = *EPgy2}Yod1*^*EY07831*^, w^*^; *P{w*^+*mC*^ = *UAS-HTT.96Q.Cerulean}2*, and *y*^*1*^* M{RFP*^*3xP3.PB*^* GFP*^*E.3xP3*^ = vas-int.Dm}ZH-2A w^*^; *M{3xP3-RFP.attP}ZH-86Fb,* and w^*^; PBac{w^+*mC*^ = *UAS-Abeta.1-42}VK00033* were from the Bloomington Drosophila Stock Center.

The following stocks: *w*^*1118*^; *P{GD11368}v21894*, *P{KK100532}VIE-260B*, *w*^*1118*^; *P{GD10916}v34574*, *w*^*1118*^; *P{GD11489}v21978*, *w*^*1118*^; *P{GD3255}v7113*, *P{KK108078}VIE-260B*, *w*^*1118*^; *P{GD7628}v18231*, *P{KK100733}VIE-260B*, *P{KK108313}VIE-260B*, *P{KK102888}VIE-260B*, *P{KK101035}VIE-260B*, *w*^*1118*^; *P{GD13944}v42609*, *w*^*1118*^; *P{GD5871}v18981*/*TM3*, *P{KK100775}VIE-260B*, *w*^*1118*^; *P{GD4739}v15340*, *w*^*1118*^; *P{GD14040}v28960*, *P{KK101867}VIE-260B*, *P{KK101319}VIE-260B*, and *P{KK100377}VIE-260B* were from the Vienna Drosophila Resource Center.

The following stocks: *w*^*1118*^; *P{RS3}not*^*CB-5509-3*^, *y*^*d2*^* w*^*1118*^* P{ey-FLP.N}2 P{5xglBS-lacZ.38-1}TPN1*; *P{neoFRT}82B P{lacW}CSN5*^*L4032*^/*TM6B*, *P{Car20y}TPN1 Tb*^*1*^ were from the Kyoto Stock Center.

Generation of *Usp5*^*2*^ deletion mutants were described in^42^. Deletion null alleles for *Yod1* and *Duba* were generated by standard P-element remobilization technique^43^. In brief, flies carrying the P-element were crossed to flies expressing the P{Δ2-3} transposase. Jump starter progeny males carrying both the P-element and the transposase were crossed to *w*; *TM3, Sb e*/*TM6B, Tb Hu e* balancer lines. Approximately 200 mutant candidate progenies were selected for each experiment and crossed to balancers to establish stocks. Candidate stocks were screened by PCR for deletions and the identified deletions were sequenced by Sanger sequencing. The origin of P-elements and the nature of mutations are in Supplementary Table S1. Indel null alleles of DUB genes were generated by an optimized CRISPR–Cas9 mutagenesis tool for *Drosophila* genome engineering^44^. For each gene, a single guide RNA targeted site was chosen in exons considering the minimal off-target effect and shortest possible distance from the START codon. Targeting sequences of 20 bp were cloned into pCFD3 guide RNA expressing vector (Addgene, cat. no. 49410). Fly stocks constitutively expressing the guide RNA were established and crossed to *nanos-Cas9* flies (Bloomington ID: 54591). G0 flies expressing both Cas9 and gRNA were crossed individually to balancer lines and four candidate progeny from each G0 line were selected and stocks were established. Approximately 100 candidate stocks for each gene were established. Insertions/deletions were identified by sequencing. The nature of mutations is on Supplementary Table S2.

To generate *UAS-Yod1.flag* lines the coding sequence of *Yod1* was amplified from genomic DNA template using Q5 DNA polymerase (New England Biolabs, NEB) and forward and reverse primers with KpnI and EcoRI restriction sites (fw: GGTACCAAAATGACGGGTTCGTTCA, rev: GAATTCGCAATCTCTCCAAAGTTCT), respectively. The amplified *Yod1* coding sequence (CDS) was cloned to pJET1.2 vector using CloneJET PCR Cloning Kit (Thermo Fisher Scientific, TFS), then subcloned to pENTR3C using KpnI, EcoRI and T4 DNA ligase (TFS). From this entry clone *Yod1* CDS was recombined to a modified, φC31 attB site containing pTWF.attB vector using Gateway LR Clonase II Enzyme mix (TFS). The pTWF.attB-Yod1 expression clone was injected to *y*^*1*^* M{RFP*^*3xP3.PB*^* GFP*^*E.3xP3*^ = vas-int.Dm}ZH-2A w^*^; *M{3xP3-RFP.attP}ZH-86Fb Drosophila* embryos for φC31 mediated site-specific transgenesis. Homozygous *w*; *UAS-Yod1-3xFLAG* transgenic strains carrying the *Yod1* construct in the third chromosomal ZH-86Fb docking site were established from F1 progeny expressing the *mini-white* marker gene.

### Viability and longevity analysis

Second or third chromosomal *DUB* mutants were crossed to *elav-GAL4*; *Sp*/*SM6b* or *elav-GAL4*; *Sb/ TM6 Hu* females, respectively, then *elav-GAL4*; *DUB*/*Sp* or *elav-GAL4*; *DUB*/*Sb* F1 male progeny were crossed to *w*; *UAS-Httex1.Q120* females in vials. Crosses were kept at 25 °C and passed once after seven days. F2 progeny were collected for five days after the beginning of eclosion and the number of flies belonging to different genotype categories were recorded. Viability was expressed as relative eclosion of the *DUB* mutation carrying mHtt expressing category ((*elav-GAL4* > *Q120 DUB*/*elav-GAL4* > *Q120*)/(*Q120 DUB*/*Q120*)).

For longevity analysis freshly eclosed *elav-GAL4* > *mHtt DUB* females and *elav-GAL4* > *mHtt* control females were transferred to fresh vials, at most 30 flies per vial. Flies were kept at 25 °C, passed to fresh vials twice a week, and the number of perished flies was recorded daily.

### Neurodegeneration assay

Degeneration of photoreceptor neurons was followed by the pseudopupil assay^18^. For this, flies were decapitated with a razor blade and their heads were fixed in a ~ 45° angle in a drop of colorless nail polish on a surface of a microscope slide. After the nail polish solidified heads were covered with immersion oil (Merck), and the structure of compound eyes was visualized using a Nikon Eclipse 80i compound microscope with 50 × oil objective. Each ommatidium of the eye of wild-type flies contains seven visible rhabdomeres, light gathering structures of photoreceptor neurons, the number of which is reduced upon degeneration of neurons. To measure the level of neurodegeneration we determined the average number of intact rhabdomers per ommatidium by counting the number of rhabdomeres in at least 20 ommatidia per compound eye of at least 10 animals per genotype.

### Climbing assay

Fruit flies exhibit negative geotaxis and climb upwards on vertical surfaces that allows the quantitation of their motor abilities. Climbing assays of groups of three-day-old female flies were performed in empty glass vials. Flies were tapped to the bottom of the vial and their upward climb was recorded on video. The distance climbed in 5 s after tapping was determined by manual curation.

### Immunohistochemistry

The salivary glands of wandering L3 larvae were dissected in PBS and mounted on microscope slides. The presence of HTT.96Q.Cerulean aggregates was visualized in at least 10 biological replicates with an Olympus Fluoview Fv10i confocal microscope using 60 × oil objective. 10–12 successive optical slices (slice thickness = 1.54 µm) were taken from each sample and the number of visible aggregates belonging to distinct size categories per cell (n_exp_ = 96, n_cont_ = 74) were determined using the ROI manager tool of ImageJ Fiji software.

### RNA-sequencing and data analysis

Total RNA was isolated from heads of 50 five-day-old females, three biological replicates per genotype (*elav-GAL4/*+, *elav-GAL4/*+; *Httex1.Q120/*+, *elav-GAL4/*+; *Yod1*^*EY07831*^*/*+, and *elav-GAL4/*+; *Httex1.Q120/*+; *Yod1*^*EY07831*^*/*+) using Monarch Total RNA Miniprep Kit (NEB). The integrity and concentration of RNA samples were determined with an Agilent 2100 Bioanalyzer instrument using Agilent RNA 6000 nano kit. PolyA-RNA samples were prepared from 800 ng total RNA with NEBNext Poly(A) mRNA Magnetic Isolation Module (NEB), then indexed, strand-specific RNA-sequencing libraries were generated using NEBNext Ultra II Directional RNA Library Prep with Sample Purification Beads (NEB) and NEBNext Multiplex Oligos for Illumina (NEB) according to the manufacturer’s protocol. Sequencing libraries were validated and quantitated with Agilent 2100 Bioanalyzer instrument using Agilent DNA 1000 kit. Equimolar amounts of indexed libraries were pooled, denatured and sequenced in three technical replicate sequencing runs in an Illumina MiSeq instrument using MiSeq Reagent Kit v3-150 kits generating 2 × 75 bp paired-end reads. Base calling and quality score generation were done by on-board Real Time Analysis (RTA 1.18.54.0) software while demultiplexing and FASTQ file generation were done by MiSeq Reporter 2.6.2.3. The average read count was 4.3 million reads per biological replicate. TrimGalore/Cutadapt was used for adapter trimming and quality based trimming of sequencing reads. Sequences were aligned to the *Drosophila melanogaster* reference genome r6.45 with HISAT2 (parameters: –rna-strandness RF)^45^ and gene specific read counts were calculated in R using the summarizeOverlaps function of Bioconductor. DESeq2^46^ was applied for differential gene expression analysis with cpm > 1 read count filter. For gene enrichment analysis FlyEnricher^20^ was used with the Human_Disease_from_FlyBase_2017 and GO_Biological_Process_2018 gene set libraries.

### Statistical analysis

We used Shapiro–Wilk test for testing normal distribution of data. In multiple comparison testing of data from the genetic screen we applied Bonferroni correction and used corrected α = 0.05 level (= 0.001428 for data shown on Fig. 1) as significance threshold. We used Χ^2^ test to analyze eclosion data. Differences in median lifespan were tested using Fischer’s exact test. Mann–Whitney U test was used to analyze unpaired samples not following normal distribution (climbing and aggregation data), pseudopupil data were analyzed using two-tailed, unpaired t-test.

### Supplementary Information

Supplementary Information.

## Data Availability

RNA-seq sequence files generated and analyzed during the current study are available in the NCBI Sequence Read Archive repository under accession PRJNA991110 (https://www.ncbi.nlm.nih.gov/sra/PRJNA991110). Other datasets used and analyzed during the current study are available from the corresponding author on reasonable request.
