# Supplementary material for: The ubiquitin thioesterase YOD1 ameliorates mutant Huntingtin induced pathology in Drosophila
Source: Sci Rep. 2023 Dec 11;13:21951. doi: 10.1038/s41598-023-49241-8 (PMC10713573; doi:10.1038/s41598-023-49241-8)
Supplement: Supplementary file 1 — Supplementary Information. [file 41598_2023_49241_MOESM1_ESM.zip › supplementary figures and tables.docx]

**SUPPLEMENTARY FIGURES**

**The ubiquitin thioesterase YOD1 ameliorates mutant Huntingtin induced pathology in *Drosophila.***

Anita Farkas^1,2^, Nóra Zsindely^1,3^, Gábor Nagy^1^, Levente Kovács^3,4^, Péter Deák^3^ and László Bodai^1,*^

^1^Department of Biochemistry and Molecular Biology, Faculty of Science and Informatics, University of Szeged, Közép fasor 52, H-6726 Szeged, Hungary

^2^Doctoral School in Biology, Faculty of Science and Informatics, University of Szeged, H-6726, Szeged, Hungary

^3^Department of Genetics, Faculty of Science and Informatics, University of Szeged, Közép fasor 52, H-6726 Szeged, Hungary

^4^Divison of Biology and Biological Engineering, California Institute of Technology, 1200 East California Boulevard, Pasadena, 91125, USA.

^*^Correspondence: bodai@bio.u-szeged.hu


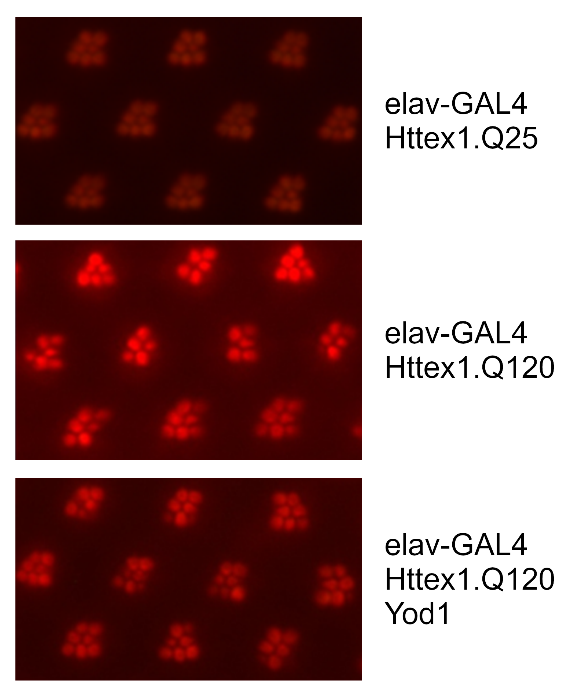


**Supplementary Figure 1.** Representative images of the retina of female flies expressing a non-pathological huntingtin fragment (Httex1.Q25), a mutant huntingtin fragment (Httex1.Q120), or co-expressing a mutant huntingtin fragment with Yod1 under the influence of the *elav-GAL4* driver. In the healthy retina, seven rhabdomeres (light-gathering structures of photoreceptor neurons) can be visualized in each ommatidium. Degeneration of photoreceptor neurons leads to loss of visible rhabdomeres.


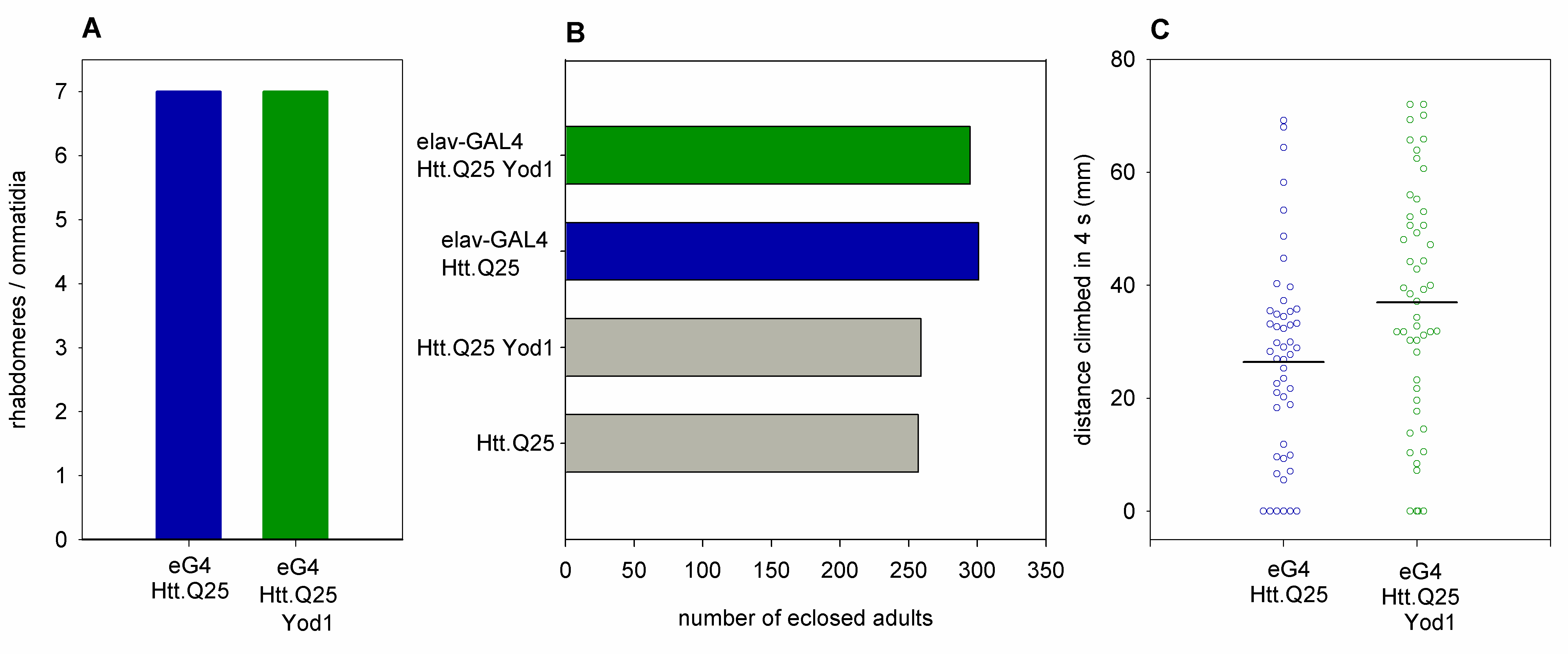


**Supplementary Figure 2.** **The effects of Yod1 in flies expressing non-pathogenic Htt.** (A) Expression of non-pathogenic Httex1.Q25, or co-expression of Httex1.Q25 and Yod1 does not induce neurodegeneration in the eyes of 7-day-old females. Bars show the average number of rhabdomeres per ommatidia in compound eyes, error bars indicate standard deviation (sd = 0 in both cases), n=10/10. (B) Neither expression of non-pathogenic Httex1.Q25, nor co-expression of Httex1.Q25 and Yod1 has a significant effect on viability. The bars show the number of eclosed adult siblings of different genotypes. (C) The climbing speed of 3-day-old flies co-expressing Httex1.Q25 and Yod1 is significantly increased compared to Httex1.Q25 expressing siblings. (P=0.008, Wilcoxon Sum of Ranks test, n_exp_=50, n_cont_=50). Datapoints show vertical distances climbed by individual flies in 4 seconds, horizontal lines show population averages.


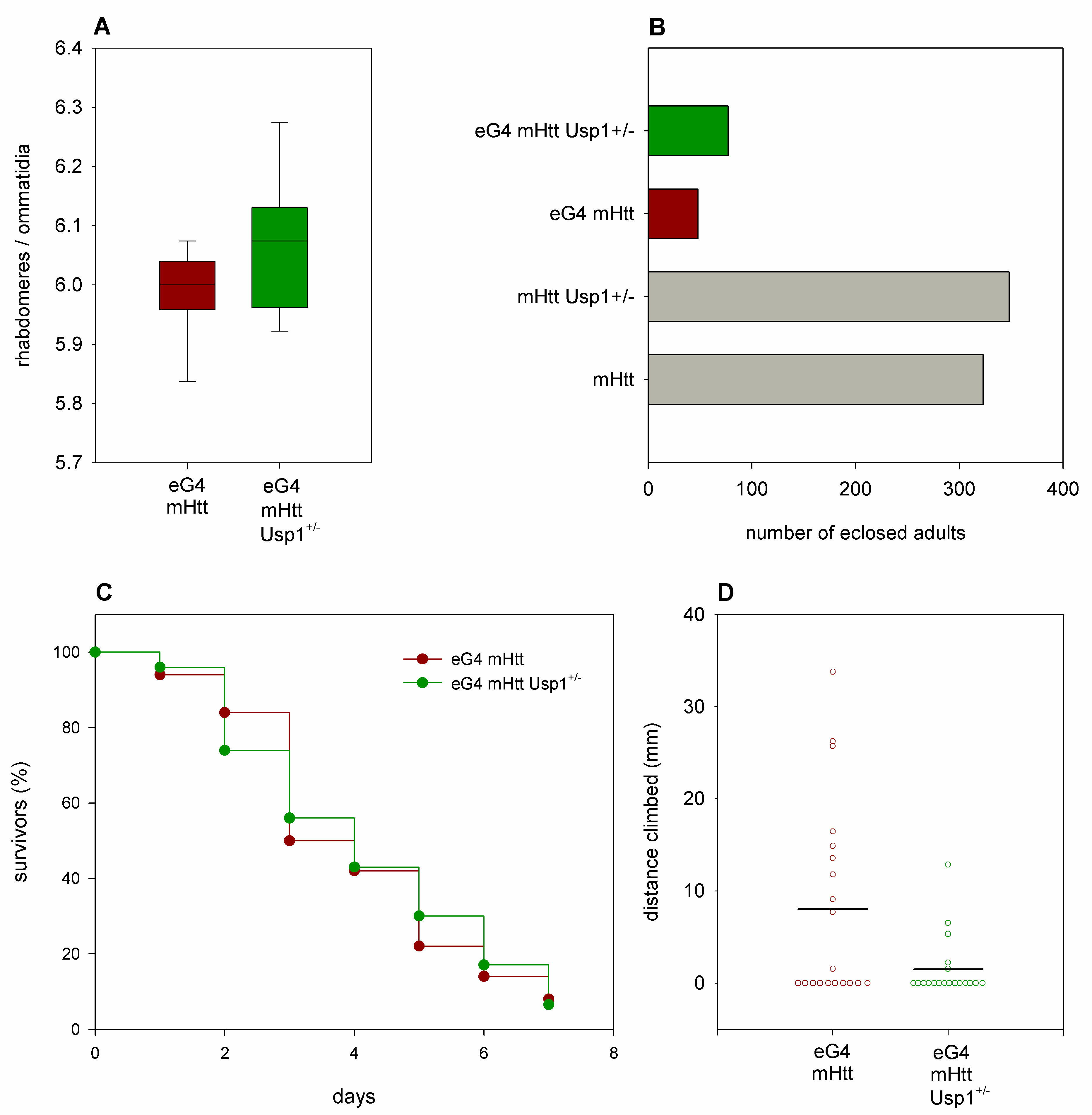


**Supplementary Figure 3.** **Reduced *Usp1* levels *via* heterozygosity of the *Usp1^c05664^* loss-of-function allele has mild, conflicting effects on mHtt-induced phenotypes.** (A) Heterozygous loss of *Usp1* in *Usp1^c05664^*/*+* mutants does not affect the degeneration of photoreceptor neurons in the eyes of 3-day-old females. Boxes show the 25^th^, 50^th^ and 75^th^ quartiles of the average number of rhabdomeres per ommatidia in compound eyes, whiskers indicate 10^th^ and 90^th^ quartile values, n=10/10. (B) Reduced levels of *Usp1* increase the number of eclosed mHtt expressing flies (P=0.045, Χ^2^-test) but (C) does not improve their median lifespan (Fisher’s exact test, n_exp_=77, n_cont_=50). (D) The climbing ability of 1-day-old mHtt expressing females heterozygous for *Usp1* is reduced compared to that of mHtt expressing control siblings (P=0.047, Wilcoxon Sum of Ranks test, n_exp_=20, n_cont_=21). Datapoints show vertical distances climbed by individual flies in 5 seconds, horizontal lines show population averages.

**

**

**Supplementary Figure 4. Overexpression of *Yod1* via the *Yod1^EY07831^* allele has mixed effects on Aβ induced phenotypes.** (A) Simultaneous neuronal overexpression of *Yod1* significantly increases the median lifespan of Aβ expressing flies (P=2.7×10^-6^, n_exp_=195, n_cont_=229), (B) but it significantly improves the degeneration of photoreceptor neurons in the eyes of 10-day-old females (P=0.0001057). Boxes show 25^th^, 50^th^ and 75^th^ quartiles of the average number of rhabdomeres per ommatidia in compound eyes, whiskers represent 10^th^ and 90^th^ quartile values, n=10/10. (C) Overexpression of *Yod1* does not have a significant effect on the climbing ability of 15-day-old Aβ expressing flies (n_exp_=94, n_cont_=79). Datapoints show vertical distances climbed by individual flies in 5 seconds, horizontal lines show population averages.


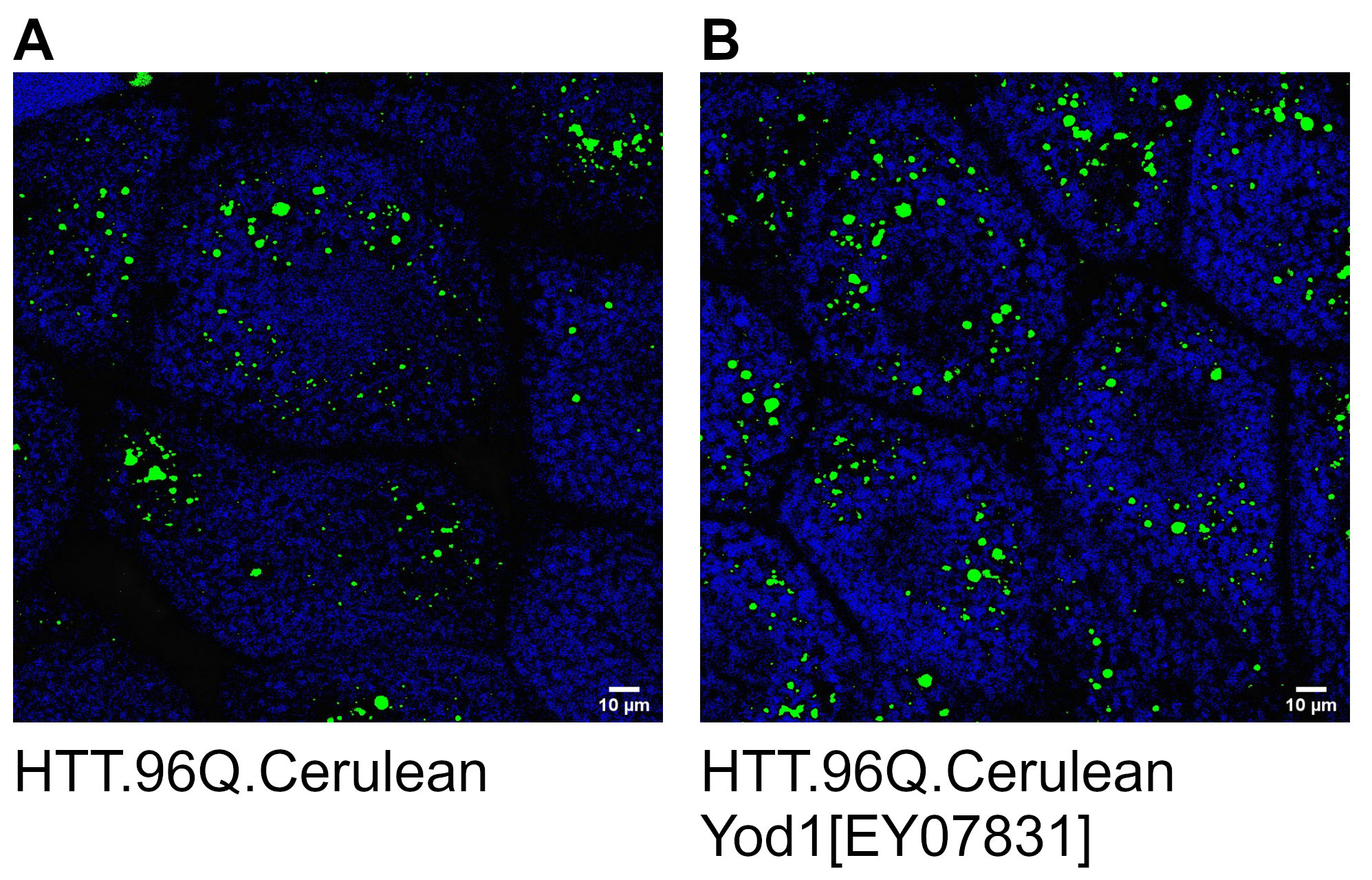


**Supplementary Figure 5.** Representative confocal images of mHtt aggregates (green) in salivary gland cells of flies expressing (A) an HTT.96Q.Cerulean transgene or (B) co-expressing HTT.96Q.Cerulean and Yod1. Background thresholds were set and artificial coloring was done using ImageJ Fiji software.

**

**

**Supplementary Figure 6. Transcript levels of Yod1 and mHtt based on RNA-seq data.** (A) The mRNA level of *Yod1* is increased in *elav-GAL4/+; Yod1^EY07831^/+* (P=0.0011, Tukey HSD after ANOVA (P=3.8×10^-5^) and *elav-GAL4/+; Httex1.Q120/+; Yod1^EY07831^/+* (P=0.0004) flies compared to *elav-GAL4/+* controls but unaltered in *elav-GAL4/+; Httex1.Q120/+* (P=0.4). (B) The expression level of the mutant Huntingtin is not decreased in *elav-GAL4/+; Httex1.Q120/+; Yod1^EY07831^/+* flies compared to *elav-GAL4/+; Httex1.Q120/+* flies rather show a statistically not significant (P=0.098) increase. Datapoints show normalized sequencing read counts of biological replicates, horizontal lines represent average values.

**Supplementary Table 1.** The origin of P-elements used for transposon-excision mutagenesis of DUB genes and the molecular nature of the generated mutations.

| **Allele** | **P element** | **Nature of allele** | **Description according to Sequence Variant Nomenclature**  **(http://varnomen.hgvs.org/)** |
| --- | --- | --- | --- |
| Yod1[Δ101/2] | P{EPgy2}Yod1[EY07831] | 1347 bp deletion eliminating the majority of the gene sequence including the START code, null | NT_037436.6.49: g. 5133801_5135148delinsN[38] |
| Duba[Δ52] | P{EP}Duba[G18421] | 2556 bp deletion eliminating the majority of the gene sequence including the START code, null | NT_037436.6.49: g. 11606338_11608894delinsN[26] |

*”delinsN” refers to the insertion of P-element footprint between the breakpoints of deletion

**Supplementary Table 2.** The molecular nature of DUB mutations generated by CRISPR–Cas9 mutagenesis.

| **Allele** | **Nature of allele** | **Description according to Sequence Variant Nomenclature**  **(http://varnomen.hgvs.org/)** |
| --- | --- | --- |
| Usp10 [CRISPR 12/2] | 2 bp deletion and 12 bp insertion in the first common exon of all three predicted transcript, frameshift and premature termination, considered null | NT_037436.6.49: g. 888904_888905del + 888960_888961insN[12] |
| Usp47 [CRISPR 28/2] | 7 bp deletion in the first exon, frameshift and premature termination, considered null | NT_037436.6.49: g. 5756294_5756300del |
| puf [CRISPR 9/2] | 1 bp insertion in exon 6, frameshift and premature termination at half way through the transcript, considered null | NT_037436.6.49: g. 24643585_24643586insA |
| Uch-L5 [CRISPR 5/1] | 4 bp deletion in exon 2, frameshift and premature termination, considered null | NT_037436.6.49: g. 9476655 _9476658del |
